# Class III β-tubulin expression as a predictor of docetaxel-resistance in metastatic castration-resistant prostate cancer

**DOI:** 10.1371/journal.pone.0222510

**Published:** 2019-10-28

**Authors:** Lucas Maahs, Bertha E. Sanchez, Nilesh Gupta, Meredith Van Harn, Evelyn R. Barrack, Prem-veer Reddy, Clara Hwang

**Affiliations:** 1 Department of Internal Medicine, Henry Ford Health System, Detroit, MI, United States of America; 2 Department of Pathology, Henry Ford Health System, Detroit, MI, United States of America; 3 Department of Public Health Sciences, Henry Ford Health System, Detroit, MI, United States of America; 4 Vattikuti Urology Institute, Henry Ford Hospital, Detroit, MI, United States of America; 5 Division of Hematology/Oncology, Henry Ford Health System, Detroit, MI, United States of America; Hunter College, UNITED STATES

## Abstract

About half of the patients treated with docetaxel in the setting of metastatic castration-resistant prostate cancer (CRPC) are non-responders. Therefore, a marker of response would be beneficial for clinical decision-making. We evaluated class III β-tubulin (βIII-tubulin) expression as a predictor of resistance in this setting, which previously has been correlated with lack of response to taxanes in other cancers. Patients with CRPC were included if they were treated with at least 3 cycles of docetaxel between 1990 and 2011. βIII-tubulin expression was assessed by immunostaining, which was performed in tissue samples obtained either via biopsy or prostatectomy at the time of diagnosis. Rates of prostate-specific antigen (PSA) response and overall survival (OS) following docetaxel treatment were compared between patients with high (2+ or 3+ staining) vs. low (0 or 1+ staining) βIII-tubulin expression. Of 73 patients, 26 (35%) had a high expression of βIII-tubulin. A PSA decline of 10% or greater occurred in 65% of patients with a high βIII-tubulin expression vs. 89% with a low βIII-tubulin expression (p = 0.0267). The median OS for patients with a high βIII-tubulin expression was 17.4 (95% CI 8.7–21.0) months vs. 19.8 (95% CI 16.6–23.6) months for patients with a low expression (p = 0.039). Our results show that a high βIII-tubulin expression is a negative prognostic factor in metastatic CRPC patients treated with docetaxel.

## Introduction

Prostate cancer is the most common solid malignancy and the second leading cause of death by cancer in men [1]. In 2016 alone, 180,890 new cases of prostate cancer were diagnosed in the United States, which corresponds to 21% of all male malignancies [1]. About 5% of these patients present with disseminated disease and thus require systemic treatment [1]. Studies have shown that between 17–30% of patients with prostate cancer treated with curative intent will have recurrence and also need systemic therapy [2–4].

The mainstay of the management of patients with metastatic prostate cancer has been androgen deprivation therapy (ADT) since the 1940s, but most patients still have disease progression, which is then defined as metastatic CRPC [5–6]. Although recent advances in the treatment of CRPC include the introduction of new drugs, such as abiraterone and enzalutamide, docetaxel remains one of the main therapeutic choices for most patients [5–6]. About half of patients receiving docetaxel for CRPC will not respond to therapy, which has propelled the search for a biomarker to predict response and aid in clinical decision-making [7]. The need for a marker is especially salient as therapeutic choices for CRPC now include docetaxel, ADT, and novel androgen-targeting therapy [5–6].

The mechanism of action of docetaxel is to stabilize microtubules, which are filamentous polymers composed of alpha- and beta-tubulin [7]. Docetaxel binds beta-tubulin, which disrupts the mitotic spindle and arrests cellular reproduction [7]. A mechanism of resistance to docetaxel is the overexpression of βIII-tubulin in tumor cells, which has been reported to correlate with a lack of treatment response in other types of cancer, such as gastric and lung cancer [8–10].

Ploussard and colleagues showed that βIII-tubulin expression was associated with a high Gleason score and an increased risk of recurrence in a sample of patients with hormone-naïve prostate cancer [11]. Continuous exposure of prostate cancer cells to docetaxel in vitro increased βIII-tubulin expression, promoting resistance to the drug. They also found an increased sensitivity to docetaxel after silencing the βIII-tubulin gene. In the same study, increased βIII-tubulin expression was associated with a shorter survival in a sample of 37 CRPC patients. To our knowledge, this has been the only study of βIII-tubulin expression in CRPC patients. We aimed to further evaluate βIII-tubulin as a marker of response to docetaxel in patients with metastatic CRPC.

## Materials and methods

### Patients

Adult men with metastatic CRPC treated with at least 3 cycles of docetaxel between 1990 and 2011 were identified retrospectively from the medical records of Henry Ford Hospital (Detroit, MI, USA). All patients were assessed to have CRPC by their primary oncologists. Only patients with available prostate cancer specimens were included in our study. Patient demographics, treatment regimens, prostate-cancer specific information (Gleason score, clinical staging, PSA, lactic acid dehydrogenase (LDH), alkaline phosphatase, hemoglobin, visceral disease, chemotherapy before docetaxel), response rates, and clinical outcomes were abstracted from the medical records. The study was approved by the Henry Ford Health System Institutional Review Board (IRB) #1 –Henry (IRB00000253) and conducted according to the principles of the Declaration of Helsinki. A waiver of consent was granted by the IRB due to our retrospective study design.

### Tissue specimen and immunostaining

Archival formalin-fixed and paraffin-embedded biopsy specimens were sectioned (4 μm thick) and attached to glass slides. For tissue microarray construction, four replicate cores with a diameter of 0.6 mm were collected from the donor block of all radical prostatectomy specimens. Immunostaining was performed with a monoclonal antibody specific for βIII-tubulin isoform (1:500 dilution, clone TUJ1; Covance, Princeton, NJ, USA). Nerve cells and axons were used as positive controls and red blood cells as negative. Scoring of immunohistochemical slides was classified according to the intensity of cytoplasmic staining in the tumor cells: no detectable stain = 0, weak stain = 1+, moderate stain = 2+, and strong stain = 3+. A high expression of βIII-tubulin was defined as at least 1% of the tumor cells having 2+ or 3+ staining, and a low expression comprised all tumor specimens having 0 or 1+ staining. A weak stain (1+) was considered negative to reduce the number of false-positives due to background staining. The pathologist was blinded to the patient’s treatment regimen, response rate and clinical outcome.

### Outcome measures

To assess how patients with high or low expression of βIII-tubulin responded to docetaxel therapy, we compared PSA levels before and after treatment. We used two cutoff points, 10% and 50%, to categorize the PSA response. We chose a ≥10% decline in PSA to evaluate any decline to reduce variation in the measurements due to chance. While a 50% cutoff is more commonly used in research to detect a more significant difference, a 10% decline in PSA is more closely related to decision-making in real clinical practice.

We used OS to measure the prognostic impact of βIII-tubulin in patients with high vs low expression. OS was defined as the period from the date of pathological diagnosis to the date of death or last follow-up.

### Statistical analysis

Comparisons of PSA response between the study groups were made using the Wilcoxon rank-sum test for continuous variables and the Fisher exact test for categorical variables. Kaplan-Meier plots and log-rank tests were used to analyze OS. The impact of βIII-tubulin on survival outcomes was calculated using the Cox proportional hazards model after adjusting for age and Gleason score. Statistical analyses were performed using SAS 9.4 (SAS Institute Inc, Cary, NC, USA).

## Results

Of 73 patients included in the study, 51% were African-American and 46% were of European descent, with a mean age of 65.7 ± 7.7 years. Table 1 displays the patient demographics and clinical characteristics at the time of prostate cancer diagnosis. Biopsy was the most common source of tissue for immunohistochemistry (77%). The median Gleason score was 8. About half of patients (52%) did not have metastasis at the time of diagnosis. The average time to metastatic CRPC was 5 years, and docetaxel was first-line therapy in 79%.

**Table 1:** Patient demographics and clinical characteristics at the time of diagnosis.

Characteristic	Number of patients	%
Race		
*African-American*	37	51
*European descent*	34	46
*Other*	2	3
Mean age, years (min-max)	65.7 (44–84.5)	
Source of tissue for Immunohistochemistry		
*Biopsy*	56	77
*Radical Prostatectomy*	17	23
Gleason score		
*Median*	8	
*4–6*	9	12
*7*	*17*	23
*8–10*	46	63
TNM staging		
*M0*	38	52
*M1*	20	27

TNM, tumor, node, metastasis; M0, no distant metastasis; M1, there is distant metastasis.

Table 2 displays the characteristics of patients with a high versus low βIII-tubulin expression at initiation of docetaxel therapy. The mean number of docetaxel cycles in the high versus low expression groups was 7.1 and 8.2, respectively. Most patients had an Eastern Cooperative Oncology Group (ECOG) performance status of <1.

**Table 2:** Clinical and pathological characteristics at the time of initiation of docetaxel therapy.

Characteristic	High βIII-tubulin	Low βIII-tubulin	p value
Number of patients (%)	26 (35)	47 (65)	
Age, years			0.056
*Mean*	68.3 ± 9.5	72.8 ± 8.0	
ECOG, performance status			0.103
*0–1*	18 (69)	38 (80)	
*2–4*	6 (23)	8 (17)	
PSA, ng/dl	428.6 ± 1072.4	399.5 ± 766.4	0.154
LDH, mg/dl	381.5 ± 439.3	273.7 ± 119.8	0.928
Alkaline phosphatase, mg/dl	216.0 ± 197.1	223.1 ± 217.7	0.931
Hemoglobin, g/dl	11.3 ± 1.8	11.3 ± 1.6	0.854
Visceral disease			0.368
*Yes*	4 (15)	4 (9)	
*No*	22 (85)	43 (91)	
Prior metastatic CRPC therapy			0.836
*No*	21 (81)	37 (79)	
*Yes*	5 (19)	10 (21)	
No. of docetaxel doses	7.1 ± 3.2	8.2 ± 3.7	0.210
Dose intensity, mg/m2*	539.3 ± 234.2	569.1 ± 275.4	0.705
TNM staging			0.276
*M0*	11 (42)	27 (57)	
*M1*	10 (38)	10 (21)	
*Mx*	5 (19)	10 (21)	
Gleason score (diagnosis)			0.225
*4–6*	1 (4)	8 (17)	
*7*	6 (23)	11 (24)	
*8–10*	19 (73)	27 (59)	

βIII-tubulin, class III β-tubulin; ECOG, Eastern Cooperative Oncology Group; PSA, Prostate-specific antigen; LDH, lactate dehydrogenase; CRPC, castration-resistant prostate cancer; TNM, tumor, node, metastasis; M0, no distant metastasis; M1, there is distant metastasis; Mx, distant metastasis cannot be assessed.

*Total dose delivered during therapy.

**Fig 1:**
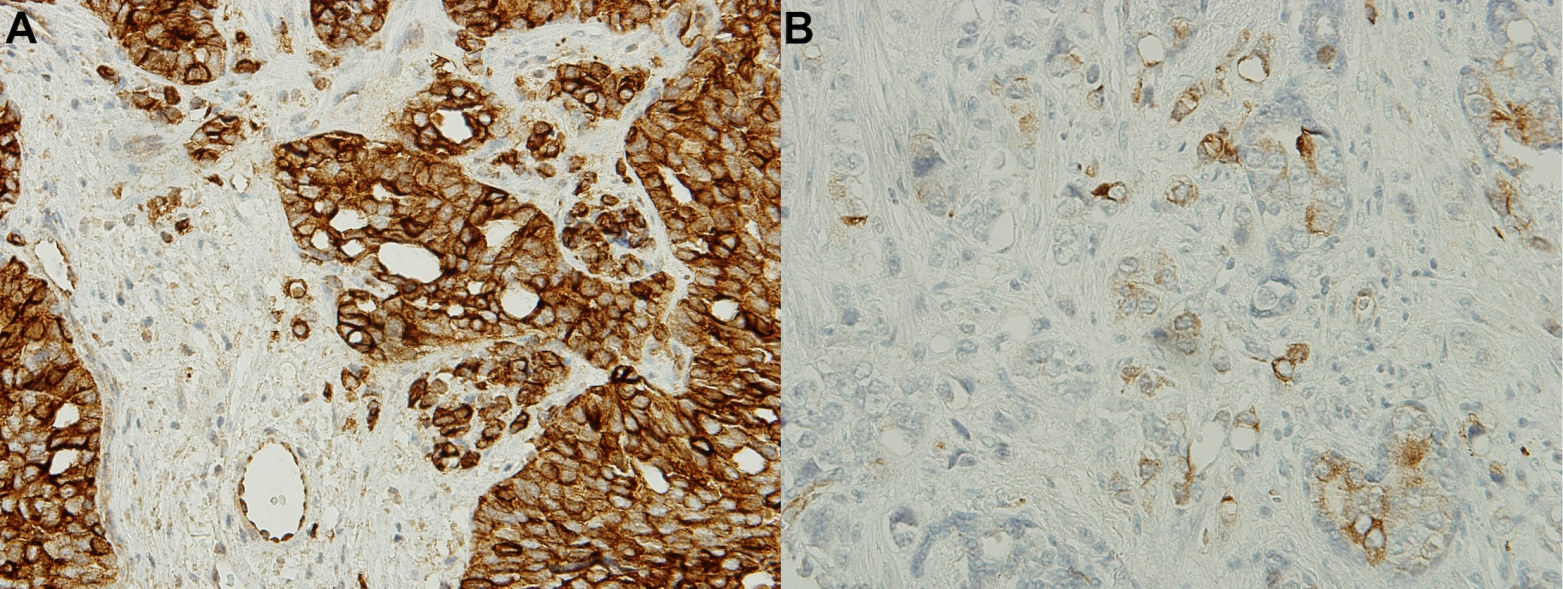
Expression of class III β-tubulin (βIII-tubulin) in available tissue samples (400x magnification). Tissue obtained via biopsy or surgery of the primary tumor was analyzed for the presence of βIII-tubulin by immunostaining. (A) Tissue with a high expression of βIII-tubulin (staining intensity 2+ or 3+). (B) Tissue with a low expression of βIII-tubulin (staining intensity 0 or 1+).

Of the 73 patients, 26 (35%) had a high expression of βIII-tubulin (Fig 1). There was no difference in the total dose of docetaxel between patients with a high versus low expression of βIII-tubulin (p = 0.705 by Wilcoxon test). The two groups also did not differ in the following clinical and pathological characteristics: age, Gleason score, tumor-node-metastasis (TNM) stage, ECOG performance status, PSA level, LDH level, alkaline phosphatase level, hemoglobin level, presence of visceral disease, and chemotherapy before docetaxel.

**Fig 2:**
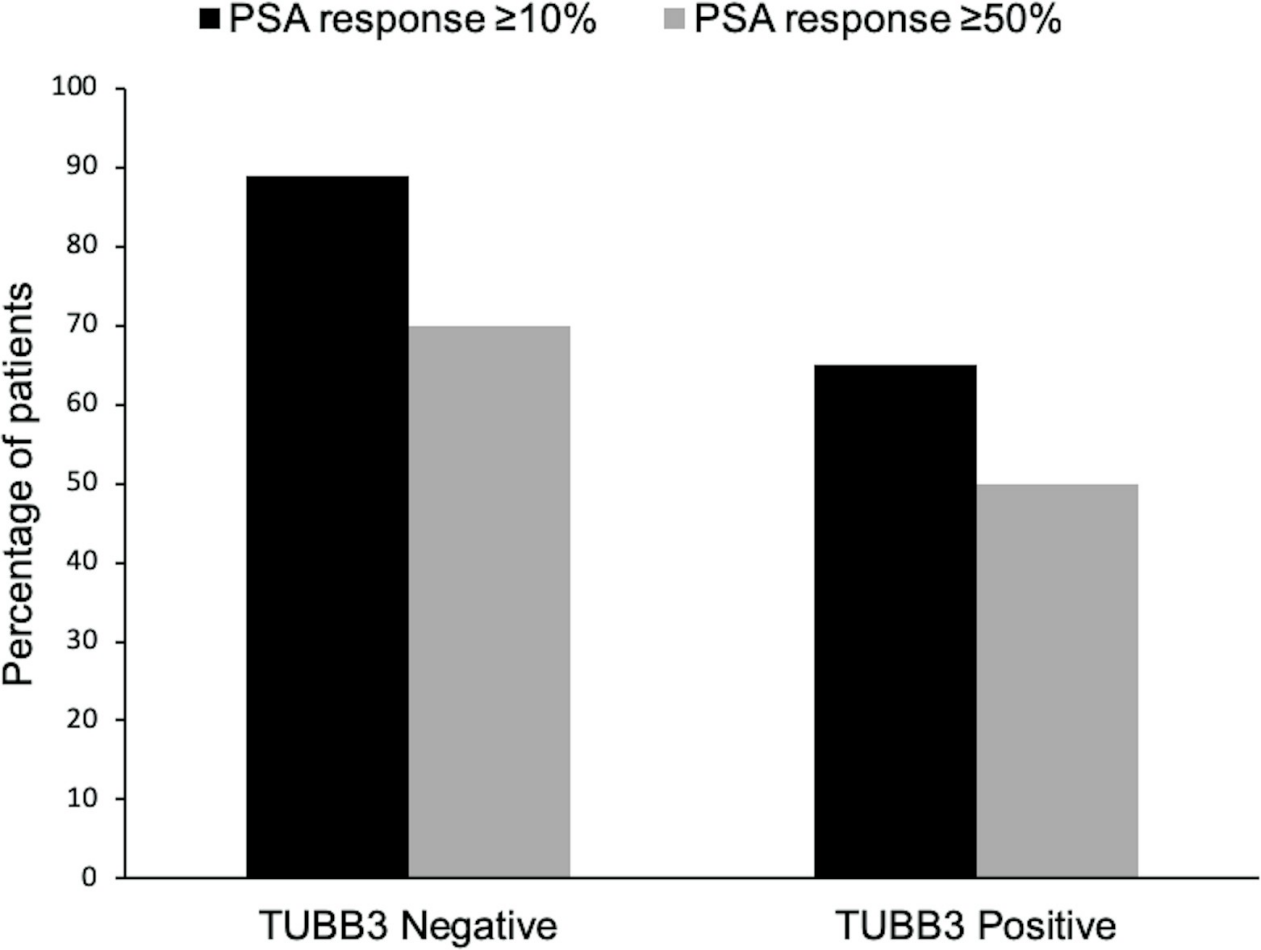
Class III β-tubulin (βIII-tubulin) expression and response to treatment with different PSA cutoffs. βIII-tubulin expression was considered high if immunostaining was moderate or strong (2+ or 3+) and low if staining was absent or weak (0 or 1+). Response to treatment was assessed via PSA measurement. Results were statistically significant when comparing percentage of patients with high vs. low expression of βIII-tubulin using the 10% cutoff (p = 0.0267).

Fig 2 shows the rates of PSA response using the two cutoff points when comparing high versus low expression of βIII-tubulin. A PSA decline of at least 10% occurend in 65% of patients with a high expression of βIII-tubulin compared to 89% of patients with a low expression (p = 0.0267). When using the 50% cutoff, 70% of the patients in the high expression group and 50% in the low expression group had a response of PSA decline, but the difference was not statistically significant (p = 0.08; Table 3). The mean PSA levels before and after treatment for each group are listed in Table 4.

**Table 3:** Response to docetaxel treatment per βIII-tubulin expression status.

Response	High βIII-tubulin		Low βIII-tubulin		p value
	No.	%	No.	%	
≥ 10% PSA decline	17	65	42	89	0.0267
≥ 50% PSA decline	13	50	33	70	0.0867

βIII-tubulin, class III β-tubulin; No., number of patients; PSA, prostate-specific antigen.

**Table 4:** Mean PSA values before and after docetaxel treatment.

Group	PSA, ng/dl (mean ± SD)		p value
	High βIII-tubulin	Low βIII-tubulin	
Before treatment	428.6 ± 1072.4	399.5 ± 766.4	0.154
After treatment—10% cutoff			
*Responders*	314.7 ± 797.5	192.2 ± 554.4	0.514
*Non-responders*	224.0 ± 336.2	579.5 ± 592.8	0.092
After treatment—50% cutoff			
*Responders*	80.8 ± 206.1	69.6 ± 215.7	0.714
*Non-responders*	468.6 ± 876.6	621.6 ± 885.4	0.269
After treatment—all patients	283.2 ± 665.4	234.0 ± 564.8	0.601

PSA, prostate-specific antigen; SD, standard deviation; βIII-tubulin, class III β-tubulin.

**Fig 3:**
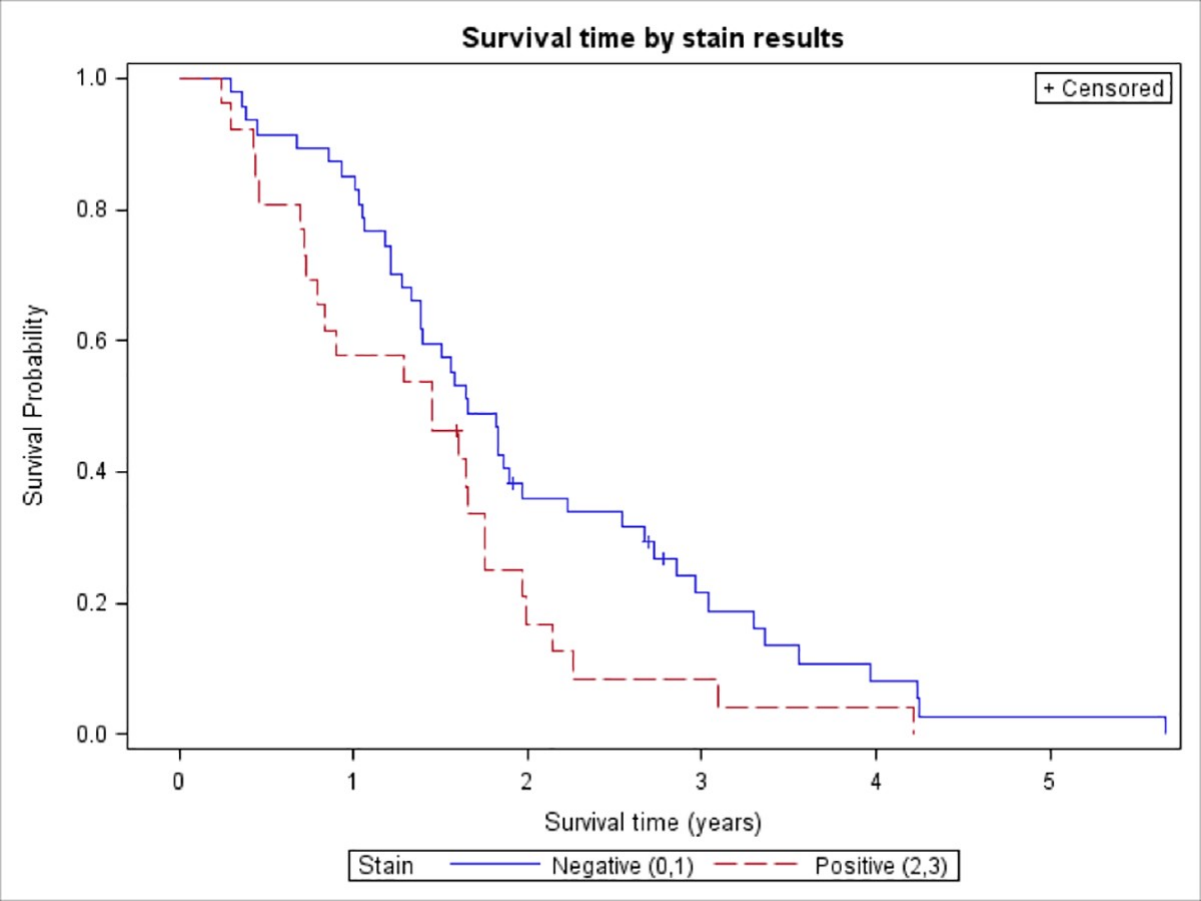
Kaplan-Meier curve of overall survival (OS) according to class III β-tubulin (βIII-tubulin) positivity. Proportion of patients that were alive over time in years from the date of pathological diagnosis. Patients were considered to have low expression if the immunostaining was absent (0) or weak (1+) and high expression if staining was moderate (2+) or strong (3+). Log-rank test of equality showed significantly longer survival time in patient with a low βIII-tubulin expression (p = 0.039). Median overall survival was 17.4 months in the high expression group and 19.8 months in the low expression group. The unadjusted HR for OS was 1.69 for the group with a high βIII-tubulin expression (95% CI, 1.02–2.80; p = 0.04).

OS for all study patients was 19.2 months (95% CI, 16.0–21.9). The median OS for patients with a high expression of βIII-tubulin was 17.4 months (95% CI, 8.7–21.0), compared to 19.8 months (95% CI, 16.6–23.6) in patients with a low expression. The log-rank test of equality of these two OS curves showed that patients with a low expression had a significantly longer survival time (p = 0.039). Fig 3 illustrates these results in a Kaplan-Meier curve.

The unadjusted hazard ratio (HR) for OS of patients with a high expression of βIII-tubulin, as compared to those with a low expression, was 1.69 (95% CI, 1.02 to 2.80; p = 0.04). A Cox proportional hazard model used covariates of Gleason score, age, and expression of βIII-tubulin to assess the influence of these predictors in the HR (Table 5). The adjusted HR for a high expression of βIII-tubulin was not statistically significant (p = 0.109).

**Table 5:** Multivariate analysis of OS.

Variable	OS			
	HR	95% CI		p value
Age >65 (vs. ≥65)	1.16	0.64	2.12	0.623
Gleason score ≤6 (vs. 4–6)	0.47	0.20	1.10	0.082
Gleason score 8–10 (vs. 4–6)	0.89	0.50	1.60	0.705
High βIII-tubulin (vs low)	1.52	0.91	2.55	0.109

OS, overall survival; HR, hazard ratio; CI, confidence interval; βIII-tubulin, class III β-tubulin.

## Discussion

This study demonstrates that metastatic CRPC patients with a high expression of βIII-tubulin had a significantly shorter survival time and worse response rates to docetaxel as indicated by a 10% or greater decrease in PSA. Our sample included a significant proportion of African-American patients (51%), which is important because this ethnic group has the highest incidence and mortality of prostate cancer [1]. The baseline characteristics of the study groups had no significant differences, reflecting a homogeneous sample. The median OS for a high expression of βIII-tubulin was 17.4 months and for low expression of βIII-tubulin was 19.8 months, which represents a clinically meaningful difference. The unadjusted HR for a high expression of βIII-tubulin was statistically significant, but the HR for high expression when adjusted to Gleason score and age was not. This may indicate that our sample size was not large enough to detect a less evident difference, given that baseline characteristics were not different between groups. We also found no statistical difference when comparing groups using a PSA cutoff of 50% (most frequently used in the literature), whereas a PSA decrease of at least 10% (most reflective of clinical practice) was significant. This finding might also be attributed to our sample size.

One limitation of our study is its retrospective nature, which precluded a formal definition for the CRPC status. Another limitation is that the tissues were obtained from the time of diagnosis and not the time of docetaxel therapy. However, some degree of correlation is still expected between samples, which was reinforced by the high expression group showing a worse prognosis. Overall, our results show that a high expression of βIII-tubulin indicates a shorter OS and a reduced treatment response with docetaxel in metastatic CRPC.

Ploussard and colleagues [11] found that βIII-tubulin expression was an independent predictor of survival in patients with CRPC undergoing docetaxel chemotherapy. However, they found no difference in response rates to docetaxel, which could be attributed to the high cutoff used for PSA decline (75%) and the small sample size. The same group of researchers [12] also suggested that a high expression of βIII-tubulin was associated with progression of prostate cancer to the castration-resistant stage. These results indicate that, regardless of the response to docetaxel, the high expression group already represents a more aggressive tumor, less likely to be controlled with ADT. Thus, besides high expression of βIII-tubulin being a marker of resistance to docetaxel, it is possible that these patients will have a worse response to any treatment and a worse prognosis overall.

The resistance to docetaxel associated with a high expression of βIII-tubulin has also been suggested in many other types of human cancers. In breast cancer, docetaxel is still one of the most used drugs for chemotherapy, and multiple studies have shown the correlation of a higher expression of βIII-tubulin with worse response to treatment [13–15]. This correlation was also demonstrated in melanoma [16], head and neck [17], ovarian [18–20], gastric [10, 21] and non-small-cell lung cancers [9, 22]. Lee and colleagues [23] showed that pancreatic cancers have a high expression of βIII-tubulin, which could be related to the high resistance of these tumors to taxanes and the poor prognosis of this type of cancer.

The mechanism of the induced resistance is not completely understood, but a few studies have provided theoretical explanations. As mentioned, as docetaxel and other taxanes bind tubulin and arrest cellular reproduction, the drug then increases the mass of polymerized tubulin, which is assembled into microtubules and leads to disruption of the mitotic spindle [7, 24]. Hari and colleagues [25] hypothesized that a high expression of βIII-tubulin results in decreased tubulin polymerization and microtubule assembly, which opposes the intended action of taxanes. Galmarini and colleagues [26] suggested two other hypotheses: a) that different classes of tubulin would confer different dynamics to the microtubules, altering sensitivity to taxanes; and b) that taxanes have a preferential binding to certain types of tubulin, and these would not include the βIII isoform.

The finding of a biomarker to select CRPC patients who might respond better to taxane therapy would be of great clinical significance. This search for a biomarker became even more important with the introduction of new first-line therapies for CRPC, such as abiraterone and enzalutamide, because patients with tumors resistant to taxanes might benefit more from these drugs. Our study suggests that βIII-tubulin expression might be an important predictor of response and potentially be a factor in clinical decision-making when selecting patients for taxane chemotherapy in the metastatic CRPC setting. However, one important consideration is that most of our patients (65%) with a high expression of βIII-tubulin responded to docetaxel treatment, which means that βIII-tubulin is unlikely to be the sole determinant in identifying which patients will benefit from docetaxel. Thus, additional markers need to be studied. Prospective studies with larger samples are also needed to better assess the value of βIII-tubulin in clinical practice.
